# Chromosome-level genome assembly of an important wolfberry fruit fly (*Neoceratitis asiatica* Becker)

**DOI:** 10.1038/s41597-023-02601-5

**Published:** 2023-10-04

**Authors:** Shaokun Guo, Bo Liu, Jia He, Zihua Zhao, Rong Zhang, Zhihong Li

**Affiliations:** 1 Department of Plant Biosecurity, College of Plant Protection, China Agricultural University, Beijing, P. R. China; 2 Ningxia Key Laboratory of Plant Disease and Pest Control, Institute of Plant Protection, Academy of Ningxia Agriculture and Forestry Science, Yinchuan, Ningxia Hui Autonomous Region P. R. China

**Keywords:** Entomology, Genome

## Abstract

Tephritidae pests are significant agricultural pests with a notable impact on the economy, with a wide range of species and most having broad host ranges and strong reproductive abilities. However, the wolfberry fruit fly, *Neoceratitis asiatica* (Becker), is a Tephritidae fly which only harms wolfberry. Here, we assembled and annotated *N. asiatica* genome at the chromosome level and compared it with the genomic and transcriptomic information from other Tephritidae flies. The assembled genome of *N. asiatica* had a size of 563.8 Mb and achieved a completeness level of 99.1%, 18,387 genes were annotated totally. All contigs were assembled into 7 linkage groups with an N50 of 93.166 Mb assisted by the Hi-C technique. The high-quality genome developed here will provide a significant resource for exploring the genetic basis of the adaptive and reproductive differences among various Tephritidae pests, and provides an important theoretical basis for the prevention and control of Tephritidae pests.

## Background & Summary

Tephritidae flies (true fruit flies) are a group of economically important insects which could damage a variety of crops comprising fruits and vegetables. They are found in all the biogeographic realms and many of them have strong invasion potential. For example, Mediterranean fruit fly (*Ceratitis capitata*) and the oriental fruit fly (*Bactrocera dorsalis*) are most important pests as far as quarantine and trade is concerned, which are responsible for millions of dollars’ worth in expenses for control and eradication, in addition to costs of damage to crops^1,2^. Interestingly, there were significant differences in host range and fecundity among different species of Tephritidae flies. *B. dorsalis* is a highly invasive pest species that damages more than 600 species of fruits, vegetables, and nut crops, a female can lay 1,000–3,000 eggs during her lifetime and there are multiple eggs in one oviposition hole^3–5^. For another notorious invasive fly, the olive fruit fly (*Bactrocera oleae*), which feeds only on the fruit of wild and cultivated olive trees (*Olea* spp.), has the capacity to ruin 100% of an olive crop by damaging the fruit. *B. oleae* can lay 200–250 eggs during the lifetime, but only one egg is produced per olive^6^. The fecundity of Tephritidae flies is affected by host and diet^7^, and Tephritidae flies which are monophagous tend to lay fewer eggs than the polyphagous flies. Nevertheless, the molecular mechanism underneath is still unclear.

The control of Tephritidae fly pests has always been a problem for researchers and farmers for a long time and the difference of host and fecundity among Tephritidae flies is a good trait to study the prevention and control techniques for this kind of pest. Genomic information provides us with a vast resource to explore the mechanisms underneath the differential performance in host preference and fecundity. From now on, 18 Tephritidae species genomes have been sequenced so far corresponding to 33 projects available at NCBI (National Center for Biotechnology Information) database (Table S1). Among the flies, most species have a wide range of hosts, only *B. oleae* and *Rhagoletis zephyria* are monophagous. More well-assembled reference genomes are essential for understanding the aspects of the ecology, evolution and adaptation of Tephritidae flies.

*Neoceratitis asiatica* Becker is a major pest on the well-known traditional Chinese medicinal plant wolfberry (*Lycium barbarum*), which is limited distributed in Ningxia, Tibet and Xinjiang, China^8^. The outbreaks of *N. asiatica* affected the yield and quality of wolfberry seriously in recent years, especially in the organic wolfberry orchards that have not been exposed to any pesticides in Ningxia, resulting in fruit-damage rates of up to 80% every year^9^. Faced with the flies that feed only on wolfberries, people are inexperienced in the prevention and control of *N. asiatica*. Similar to *B. oleae*, *N. asiatica* lays one egg per fruit normally, occasionally 2 or 3 eggs are laid but only one larva survives^10,11^.

In the present study, the genome of *N. asiatica* was de novo assembled based on sequences obtained from Nanopore and Illumina platforms and then assembled at the chromosome-level assisted by the Hi-C (High-throughput chromosome conformation capture) technique. This novel genomic resource allowed us to compare genomic changes in the evolution of Tephritidae flies for exploring the reasons of host diversity and reproductive differences. The contraction of detoxification and chemosensory gene families may contribute to the limited host and fecundity in *N. asiatica*. Based on the genome and transcriptome, we have investigated the differentially expressed genes and revealed molecular process from host identification to reproduction and oviposition. Overall, the *N. asiatica* genome provides a useful resource for understanding the genetic basis of relationship between fecundity and host range in Tephritidae flies more generally, and also facilitates the selection of more potential controlling target genes, which may ultimately be useful for the management of the Tephritidae pests.

## Methods

### Samples and DNA preparation

*Neoceratitis asiatica* were collected from wolfberry orchard in Ningxia for genome sequencing. Genomic DNA used for the Nanopore and the Illumina paired-end library preparation was extracted from 10 thoraxes with QIAGEN® Genomic kit (Cat#13343, Qiagen, Hilden, Germany), following the manufacturer’s instructions. All DNA extracts were verified with NanoDrop (NanoDrop products, Wilmington, DE, USA) and a Qubit 3.0 Fluorometer (Life Technologies Corporation, Eugene, OR, USA) using the Qubit^TM^ dsDNA HS Assay Kit (PN# Q32851) (Life Technologies Corporation, Eugene, OR, USA) to quantify the purity and concentration.

### Library construction and sequencing

The BluePippin automatic nucleic acid recycling instrument (Sage Science, Beverly, MA, USA) was used to cut and recycle large fragments, then the fragmented DNA was repaired and purified using NEBNext FFPE Repair Mix (New England BioLabs, NEB, Ipswich, MA, USA). The end repair and a ligation were carried out using NEBNext End repair/dA-tailing Module and NEBNext Quick Ligation Module (NEB, Ipswich, MA, USA). After purification, the adaptors were linked using the Ligation Sequencing Kit (Cat# SQK-LSK109, Aberdeen) (Oxford Nanopore Technologies, Oxford, UK). Qubit®3.0 Fluorometer (Life Technologies Corporation, Eugene, OR, USA) was used for accurate quantitative examination of the established DNA libraries. Approximately 700 ng DNA library was constructed and performed on a Nanopore PromethION sequencer instrument (Oxford Nanopore Technologies, Oxford, UK) for real-time single-molecule sequencing at the Genome Center of Grandomics (Wuhan, China). For short-read sequencing, a paired-end library construction and sequencing were carried out as described in previous publication^12^. After filtering, we obtained 31.460 Gb of short clean reads from the Illumina platform (coverage: 55.799 X) and 74.136 Gb raw data (coverage: 131.493 X) from the Nanopore platform for contig-level genome assembly (Table S2).

### Genome de novo assembly and evaluation

The Oxford Nanopore long-reads were used for genome de novo assembly. Raw reads were corrected and assembled using NextDenovo v2.4.0 (https://github.com/Nextomics/NextDenovo) with parameters of “ctg_cns_options: -p 30; nextgraph_options: -a 1; sort_options: -m 50g -t 30 -k 40; minimap2_options_map: -x map-ont; minimap2_options_raw: -t 8 -x ava-ont” to generate a draft assembly. After assembly, the NextPolish v1.3.1^13^ was used to further improve single base accuracy with default parameters (sgs_options = -max_depth 100 -bwa; lgs_options = -min_read_len 1k -max_depth 100). The Oxford Nanopore long reads were assembled into 198 contigs, with a contig N50 length of 19.202 Mb (Table S2). After mapping the Illumina reads to the reference genome using Burrow-Wheeler Aligner (BWA) v0.7.17^14^, the coverage was calculated. Genome size, heterozygosity, and duplication of the genome were estimated by the K-mer method. K-mers were counted by jellyfish v2.2.9^15^ with 17-base and 21-base oligonucleotide based on Illumina short reads (Fig. S1a,b). Parameters were determined by GenomeScope v1.0^16^. Benchmarking Universal Single-Copy Orthologs (BUSCO) v4.1.4 was used to evaluate the completeness of the assembly based on the insecta_odb10 database (1,367 genes)^17^, which showed that 99.3% (single-copy gene: 98.8%, duplicated gene: 0.5%) and 99.1% (single-copy gene: 98.7%, duplicated gene: 0.4%) were identified as complete from contig- and chromosome-level genome, respectively (Table S3). We also compared the genome assembly features among 10 fruit flies (Table 1).

**Table 1:** Assembly features for genomes of *Neoceratitis asiatica* and other insect species.

Feature	***N. asiatica***	***C. capitata***^53^	***B. dorsalis***^54^	***B. latifrons***^55^	***B. tryoni***^56^	***B. oleae***^57^	***Z. cucurbitae***^58^	***R. zephyria***^59^	***R. pomonella***^59^	***D. melanogaster***^60^
Level	Chr.	Scaf.	Chr.	Scaf.	Chr.	Scaf.	Scaf.	Scaf.	Scaf.	Chr.
Size (Mb)	563.8	436.5	468.7	462.5	570.6	484.9	374.8	1,110	1,223	143.73
No. scaffold/chromosome	7	2,354	6	3,305	5	38,160	5,571	84,794	32,060	7
Scaffold N50 (Mb)	93.166	1.7	90.5	0.9744	81.9	4.6	1.4	0.063	72.3	24.116
No. contig	198	3,242	6	30,468	8,397	48,617	43,001	135,237	114,121	2,442
Contig N50 (Mb)	19.202	0.8459	90.5	0.0315	0.3509	0.1877	0.0174	0.0194	0.0236	20.490
Completeness (%)	99.1	99.7	99.78	99.3	99.2	98.8	99.4		94.8	99.7
No. gene	18,387	14,236	15,775	14,128	16,748	16,147	14,663	28,476	25,184	17,468

### Hi-C libraries and genome scaffolding

**Fig. 1:**
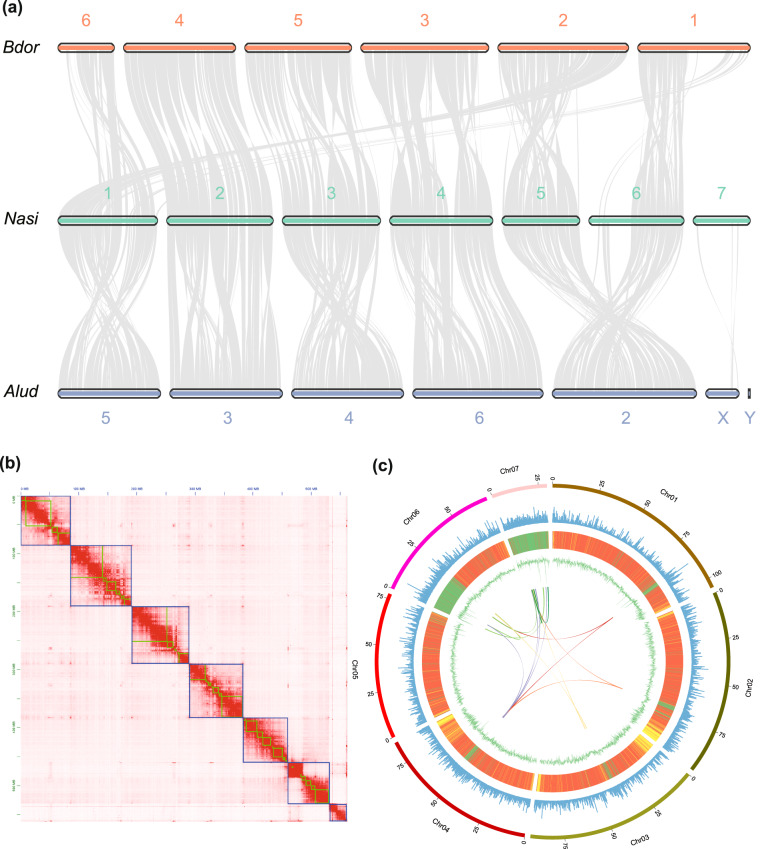
The visualization of *Neoceratitis asiatica* genomic details resulting from high-quality assembly. (**a**) Synteny blocks among *Neoceratitis asiatica* (*Nasi*), *Bactrocera dorsalis* (*Bdor*), and *Anastrepha ludens* (*Alud*) genomes; (**b**) Genome-wide all-by-all Hi-C interaction, only sequences anchored on chromosomes are shown in the plot, one green box indicates one contig and the blue box indicates the chromosome; (**c**) Circular diagram depicting the characteristics of the *Neoceratitis asiatica* genome. The outer layer of coloured blocks is a circular representation of the 7 linkage-groups and circos demonstration of gene count (histogram), repeat density (heatmap) and GC content (line) from the outer to the inner circle, respectively. The coloured arcs are shown as the intra-genomic link.

The Hi-C technique was applied to capture genome-wide chromatin interactions for assisting the chromosome-level assembly^18^. Thoraxes from 10 males of *N. asiatica* were ground in 2% formaldehyde to allow cross-linking of cellular protein, cross-linking was then stopped by adding glycine and additional vacuum infiltration. Fixed tissue was then grounded to powder before re-suspending in nuclei isolation buffer, then the purified nuclei were digested with 100 units of DpnII restriction enzyme and marked by incubating with biotin-14-dATP. Biotin-14-dATP from non-ligated DNA ends was removed owing to the exonuclease activity of T4 DNA polymerase. The ligated DNA was then blunt-end repaired and A-tailed, followed by purification through biotin-streptavidin-mediated pull down. Hi-C libraries were quantified and sequenced on the Illumina NovaSeq platform, generating 150 bp paired-end reads. In total, we generated 51.876 Gb (92.011 X coverage) of Hi-C data for *N. asiatica* (Table S2). Juicer v1.6 (mapq threshold >30) and 3D de novo assembly (3D-DNA) pipelines were used to assemble the scaffolds into a chromosome-level genome^19,20^. There were 49.58% normal paired reads while the others were chimeric paired (39.55%), chimeric ambiguous (9.83%) or unmapped reads (1.04%), and 45.48% of the read pairs showed Hi-C contacts (Table S4). The assembled contigs were clustered into 7 linkage groups with an N50 of 93.166 Mb (Fig. 1b, Table 1).

### Chromosomal synteny analysis

To compare structural characteristics of the genomes among fruit flies, we analysed chromosomal synteny between fruit flies based on genome-scale ortholog alignment using the JCVI, which is the python version of MCScanX (Multiple Collinearity Scan toolkit)^21^. We chose 7 Tephritidae flies for the synteny analysis and found extensive chromosome synteny between *N. asiatica* and other flies (Fig. 1a & Fig. S2a,b). Most chromosomes of *N. asiatica* were structurally unchanged in comparison with chromosomes of other fruit flies and some originated by fission of an ancestral chromosome, such as chromosome 5 and 6. Due to the lack of chromosomal genome of the most closely related species *Ceratitis capitata*, we analysed the synteny using 1:1 orthologs genes and found that there’re well synteny between *N. asiatica* and *C. capitata* (Fig. S2c). In addition, MUMmer was used for the alignment of large fragment sequences between *N. asiatica* and *C. capitata*, as well as between *N. asiatica* and *B. dorsalis*^22^. There was also a higher similarity between the *N. asiatica* and *C. capitata* at genomic level (Fig. S3a), while there’re significant differences in the chromosome structure between *N. asiatica* and *B. dorsalis* (Fig. S3b). A circular diagram representing gene count, repeat density and GC content was generated using Circos^23^, which were shown in a circular diagram (Fig. 1c).

### Genome annotation

Gene structure annotation was conducted in Maker v3.01.03 genome annotation pipeline as described in previous publication^12,24^. The software eggnog-mapper v2.1.7 was applied to annotate gene functions^25^. We identified 18,387 annotated proteins and BUSCO analysis showed that 90.7% (single-copy gene: 90.0%, duplicated gene: 0.7%) of the evaluated single-copy genes were identified as complete, 3.1% of the genes were fragmented, while 6.2% of the genes were missing in the gene set (Table S3). Repetitive elements in scaffolds longer than 1,000 bp were detected by RepeatMasker v4.0.7^26^ against the Insecta repeats within RepBase Update (http://www.girinst.org). The assembled genome was analyzed for potential DNA transposon sequences using the program RepeatModeler (http://www.repeatmasker.org/RepeatModeler.html, RRID: SCR_015027). The noncoding RNAs (ncRNA) including transfer RNA (tRNA), ribosome RNA (rRNA), microRNA (miRNA), small nuclear RNA (snRNA) and small nucleolar RNA (snoRNA) were annotated by aligning the genomic sequence against RFAM (http://rfam.xfam.org/). Among them, tRNAs and rRNAs were predicted by tRNAscan-SE and RNAmmer with default parameters^27,28^, other ncRNA (miRNA, snRNA, snoRNA) were annotated by cmscan program^29^. In total, 86 rRNAs, 1,368 tRNAs, and 69 micro RNAs were predicted in the *N. asiatica* genome (Table S5). There’re 222,258,971 bp (39.6%) transposable elements (TEs), including 371,986 retroelements and 264,341 DNA transposons. Totally 64 satellites and 283,449 simple repeats were identified as tandem repeats (TRs), accounting for 0.01% and 3.61% of the *N. asiatica* genome, respectively (Table S6).

### Comparative genome analysis

**Fig. 2:**
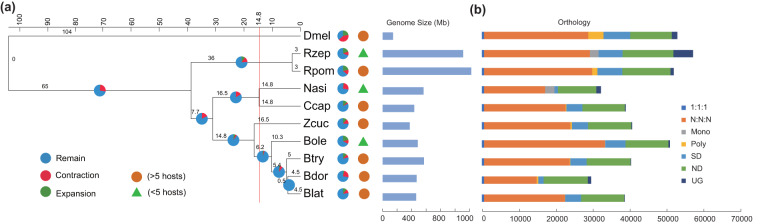
Phylogenetic and genomic comparisons of 10 fruit flies. (**a**) Phylogenetic position of *Neoceratitis asiatica* relative to other insects. Branch lengths of the tree are scaled to estimated divergence time in Mya with every million years presented by dotted lines. Tree topology is supported by posterior probabilities of 1.0 for all nodes. The red line shows the divergence time between *Neoceratitis asiatica* and *Ceratitis capitata*. The pie charts of significantly expanded (green), contracted (red) and remained (blue) gene families are labelled following each branch. Host range and genome size are also shown here. Orange circles represent species with more than five hosts, and green triangles represent species with fewer than five hosts. (**b**) Bar plots show total gene counts for each fly partitioned according to their orthology profiles, including single-copy genes in all species (1:1:1), multi-copy genes in all species (N:N:N), monophagous fly-specific genes (Mono), polyphagous fly-specific genes (Poly), species-specific duplicated genes (SD), species-specific genes (ND) and unassigned genes (UG).

Identification of orthology and phylogenetic relationships construction were carried out as described in previous publication^12^. Orthologues and orthogroups were identified using OrthoFinder^30^. Phylogenetic relationships within 10 fruit flies were reconstructed based on single-copy orthologs of protein-coding genes, there’re 3 monophagous flies (*N. asiatica*, *B. oleae* and *R. zephyria*), 6 polyphagous flies (*C. capitata*, *B. dorsalis*, *Bactrocera latifrons*, *Bactrocera tryoni*, *Zeugodacus cucurbitae* and *Rhagoletis pomonella*), and the fruit fly *D. melanogaster* as the outgroup. The phylogenetic tree was inferred using an approximately-maximum-likelihood method implemented in FastTree version 2.1.10^31^ under default settings. The resulting ML tree was used as an input tree for the Cafe5 which was used to identify the gene family that had undergone expansions or contractions for the gene families with among 10 insect genomes^32^, and the tree was further optimized by iTOL (Interactive Tree of Life) server^33^. The tree supported the sister relationship between *Bactrocera* and *Zeugodacus*, and the monophyly of *Rhagoletis* (Fig. 2a), congruent with currently accepted topologies of Tephritidae^34,35^. OrthoFinder assigned 246,374 genes (95.6% of total) to 17,735 orthogroups for 10 fruit flies (Fig. 2b, Tables S7, S8). Fifty percent of all genes were in orthogroups with 18 or more genes (G50 was 18) and were contained in the largest 3,777 orthogroups (O50 was 3,777). There were 7,695 orthogroups with all species present and 613 of these consisted entirely of single-copy genes. We detected expansion of 439 families and contraction of 4,527 families in *N. asiatica* genome (Fig. 2a and Table S9), which has the lowest expansion ratio.

**Fig. 3:**
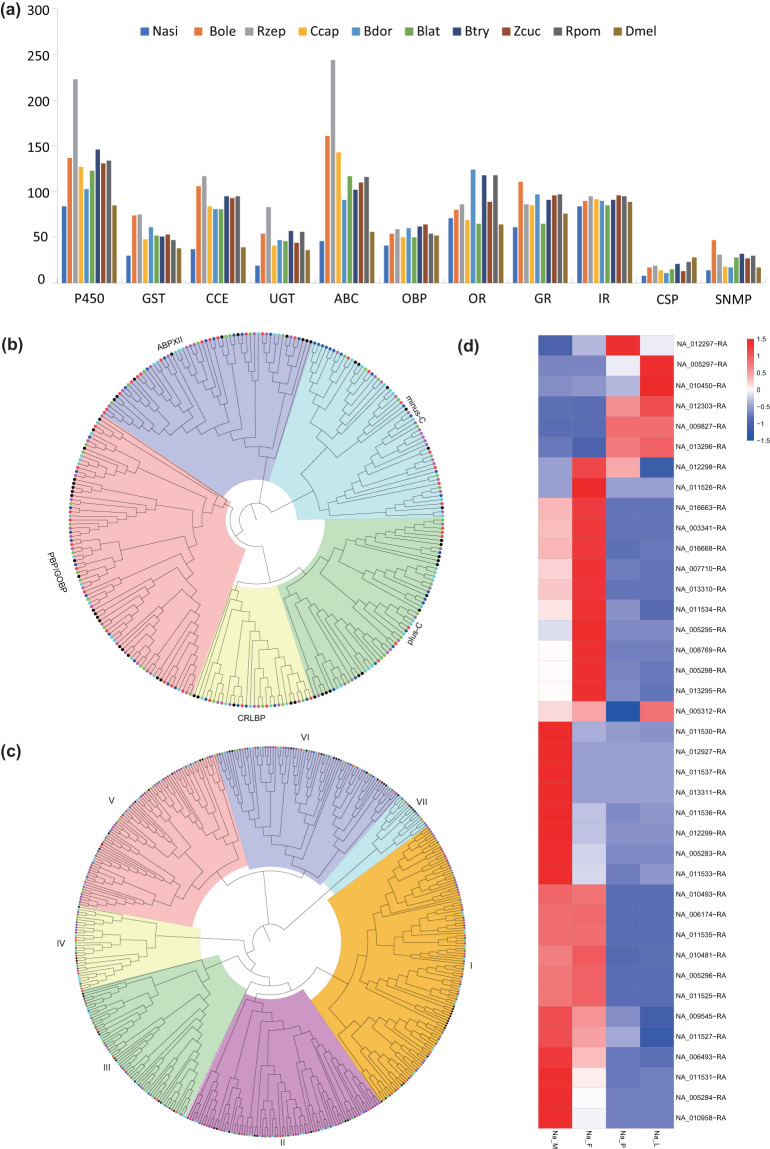
Evolution of detoxification and chemosensory genes. (**a**) Histogram of the number of genes in five detoxification families (P450, GST, CCE, UGT and ABC) and six chemosensory families (OBP, OR, GR, IR, CSP and SNMP). (**b**) Phylogenetic tree of *Neoceratitis asiatica* OBP genes in comparison with other insects. (**c**) Phylogenetic tree of *Neoceratitis asiatica* OR genes in comparison with other insects, I to VII represent 7 subfamilies of OR gene families. (**d**) Expressed differences of OBP genes at different developmental stages in *Neoceratitis asiatica*. Na_M, newly emerged males of *Neoceratitis asiatica*, Na_F, newly emerged females of *Neoceratitis asiatica*, Na_P, 2-day-old pupae of *Neoceratitis asiatica*, Na_L, Mature larvae of *Neoceratitis asiatica*. Purple dot, *Bactrocera dorsalis*; Green dot, *Bactrocera oleae*; Dark blue dot, *Ceratitis capitata*; Black dot, *Drosophila melanogaster*; Red dot, *Neoceratitis asiatica*; Light blue dot, *Rhagoletis zephyria*.

Eleven gene families covering detoxification and chemosensory genes were manually annotated, including cytochrome P450 monooxygenase (P450s), glutathione S-transferase (GSTs), carboxyl/cholinesterase (CCEs), UDP-glycosyltransferases (UGTs), ATP-binding cassette (ABC) transporter, odorant binding protein (OBP), odorant receptor (OR), gustatory receptor (GR), Ionotropic receptors (IR), chemosensory proteins (CSP), and sensory neuron membrane protein (SNMP). The bioinformatic pipeline BITACORA (full mode) was used to conduct HMMER and BLAST analyses^36^. The annotated genes were filtered with a default cutoff E-value of 10e-5 and then manually based on gene length and the presence of conserved domains. Orthologs were aligned with the G-INS-I algorithm implemented in MAFFT v7.450^37^. A neighbor-joining tree was constructed for each gene family using MEGA7^38^ with 1000 bootstrap replicates. We identified 84 P450s, 30 GSTs, 37 CCEs, 19 UGTs and 46 ABC transporters in the *N. asiatica* genome (Fig. 3a, Table 2). There’re 279 chemosensory genes in *N. asiatica*, including 41 OBPs, 71 ORs, 61 GRs, 84 IRs, 8 CSPs and 14 SNMPs. The subfamilies of OBPs were further analyzed among different flies, the PBP/GOBP and plus-C subfamilies comprised a relatively few numbers of genes in *N. asiatica* as compared to the other subfamilies (Fig. 3b). For ORs, *N. asiatica* had lower numbers of genes in comparison to *B. dorsalis*, *B. tryoni* and *R. pomonella*, but had no significant difference with the other two monophagous flies (*B. oleae* and *R. zephyria*), particularly in the group I, II, and V (Fig. 3c). Overall, comparing with other fruit flies, *N. asiatica* had less detoxification and chemosensory genes.

**Table 2:** Statistics on detoxification, chemosensory genes across *Neoceratitis asiatica* and other insects.

Common name	Species code	P450	GST	CCE	UGT	ABC	OBP	OR	GR	IR	CSP	SNMP
Monophagous flies	*N. asiatica*	84	30	37	19	46	41	71	61	84	8	14
	*B. oleae*	137	74	106	54	161	40	71	75	90	17	27
	*R. zephyria*	223	75	117	83	244	48	79	86	95	19	31
Polyphagous flies	*C. capitata*	127	48	84	41	143	47	69	85	92	14	18
	*B. dorsalis*	103	61	81	47	91	59	122	97	90	11	17
	*B. latifrons*	123	52	81	46	117	50	65	65	85	15	28
	*B. tryoni*	146	51	95	57	102	62	118	91	91	21	32
	*Z. cucurbitae*	131	53	93	44	110	64	89	96	96	13	27
	*R. pomonella*	134	47	95	56	116	54	118	97	95	23	30
	*D. melanogaster*	85^61^	38^62,63^	39^64^	36^65^	56^66^	51	62	76	89	28	17

### RNA-seq and analyses

Mature larvae, 2-day-old pupae, newly emerged males and females of *N. asiatica* were collected at the same time of day for transcriptome sequencing and gene expression analysis. Total RNA was isolated from samples collected above with TRIzol Reagent (Invitrogen, Carlsbad, CA, USA) and quantified with a NanoDrop ND-2000 spectrophotometer (NanoDrop products, Wilmington, DE, USA). Three biological replicates, each containing 1 individual, were set up for each developmental stage. TruSeq^TM^ RNA sample preparation Kit from Illumina (San Diego, CA, USA) was used for cDNA libraries construction using 1 μg of total RNA. Libraries were size selected for target fragments of 300 bp on 2% low range ultra-agarose followed by PCR amplified for 15 cycles using Phusion DNA polymerase (NEB, Ipswich, MA, USA). After quantified by TBS380 (Picogreen), paired-end library was sequenced with the Illumina NovaSeq 6000 sequencer (Illumina, San Diego, CA, USA) at the Majorbio Bio-pharm Technology Co., Ltd (Shanhai, China). The analyses were performed with the online platform of the Majorbio I-Sanger Cloud Platform (www.i-sanger.com). The analyses of gene expression profiles across all developmental time points showed that the large number of differentially expressed genes (DEGs) between mature and immature stages (Fig. S4). The male stage had the largest number of up-regulated expressed genes (3871), whereas 3088, 2445 and 2381 up-regulated expressed genes were selected in the female, pupal and larval stages, respectively, and the results were deposited at figshare^39^. There’re more OBP genes up-regulated expressed in *N. asiatica* adults, including OBP57c, 99a, 99b, 69a, 19a, 19d, 84a and 56d in females, and OBP57c, 99a, 69a, 56h, 56d, 19a and 19d in males (Fig. 3d). Odorant detection associated adenylate cyclase 3 (ADCY3) was only significant up-regulated expressed in adult stage.

For the transcriptomic analysis with multiple species, we downloaded the RNA-seq raw data of other fruit flies from the SRA repository^40–43^. After trimming both adapters and low-quality reads (Phred quality score <30) using Trimmomatic (v.0.35)^44^, the clean reads were mapped to the respective genome using STAR v2.6.0c with default parameters^45^. Read counts were calculated with RSEM v1.2.9^46^ and DEGs between two species in the same developmental stages were analysed using Edge R^47^ with trimmed mean of M-values (TMM) normalization^48^. In this analysis, we adopted the well-established Benjamini-Hochberg method to calibrate *p* values from the original assumption test^49^. After calibration, the *p* value was determined using the false discovery rate (FDR) approach to decrease false positives caused by independent statistical hypothesis testing on expression changes in a large number of genes. We used an FDR <0.05 and a |log (fold-change (FC)) | ≥ 1 as the criteria for a significant difference in expression. DEGs were mapped to GO terms and KEGG pathways, and an enrichment analysis was performed to identify any over-representation of GO terms and KEGG pathways.

## Data Records

The genome sequence and gene sequence had been deposited at the National Center for Biotechnology Information (NCBI), under the accession number of JANTOX000000000^50^. The NCBI BioProject accession number is PRJNA869884. Raw reads obtained for genome assembly have been deposited in the Sequence Read Archive (SRA) repository with the accession number of SRP392573^51^. In addition, the genome annotation files had been submitted at the figshare^52^.

## Technical Validation

The integrity of the extracted DNA was checked by agarose gel electrophoresis, and the concentration of DNA was determined using NanoDrop (NanoDrop products, Wilmington, DE, USA) and Qubit 3.0 Fluorometer (Life Technologies Corporation, Eugene, OR, USA) with an absorbance of approximately 1.80 at 260/280. Scaffold N50 (the length such that half of all sequence is in scaffolds of this size) has achieved a significant improvement to 93.166 Mb, which is much higher than other genomes (Table 1). We used the sequence identity method to evaluate the completeness of the genome assembly, selected small fragment library reads, and used BWA software to align them with the assembled genome. The completeness (99.1%) estimated using BUSCO also provides confidence in the quality of the assembled genome (Table 1, Table S3). The proportion of the chromosome-level genome involving duplicated single-copy genes evaluated in BUSCO was very low (0.4%) (Table S3), indicating that duplication was not a major issue in assembling the genome. These results showed that we obtained the high-quality genome of *N. asiatica*.

### Supplementary information

Supplemental Information

## Data Availability

The data analyses were performed according to the manuals and protocols by the developers of corresponding bioinformatics tools and all software, and codes used in this work are publicly available, with corresponding versions indicated in Methods.
