# Supplementary material for: Chromosome-level genome assembly of an important wolfberry fruit fly (Neoceratitis asiatica Becker)
Source: Sci Data. 2023 Oct 4;10:675. doi: 10.1038/s41597-023-02601-5 (PMC10551018; doi:10.1038/s41597-023-02601-5)
Supplement: Supplementary file 1 — Supplemental Information [file 41597_2023_2601_MOESM1_ESM.docx]

**Supplemental Information for:**

**Chromosome-level genome assembly of** **an important wolfberry fruit fly (*Neoceratitis asiatica* Becker)**

Shaokun Guo ^1, *^, Bo Liu ^1^, Jia He ^2^, Zihua Zhao ^1^, Rong Zhang ^2^, Zhihong Li ^1, *^

1. Department of Plant Biosecurity, College of Plant Protection, China Agricultural University, Beijing, P. R. China

2. Ningxia Key Laboratory of Plant Disease and Pest Control, Institute of Plant Protection, Academy of Ningxia Agriculture and Forestry Science, Yinchuan, Ningxia Hui Autonomous Region, P. R. China

*** Corresponding authors**:

**Shaokun Guo**, [guoshaokun@cau.edu.cn](mailto:guoshaokun@cau.edu.cn)

**Zhihong Li**, [lizh@cau.edu.cn](mailto:lizh@cau.edu.cn)

**Table of Contents:**

| **Table S1** | Page 3 |
| --- | --- |
| **Table S2** | Page 5 |
| **Table S3** | Page 5 |
| **Table S4** | Page 6 |
| **Table S5** | Page 7 |
| **Table S6** | Page 8 |
| **Table S7** | Page 9 |
| **Table S8** | Page 10 |
| **Table S9** | Page 11 |
| **Figure S1** | Page 12 |
| **Figure S2** | Page 13 |
| **Figure S3** | Page 14 |
| **Figure S4** | Page 15 |

**Table S1** **The genomic information of Tephritidae from NCBI**

| Assembly Accession | Assembly Name | Organism Name | Assembly Stats Total Sequence Length | Assembly Level | Assembly Submission Date |
| --- | --- | --- | --- | --- | --- |
| GCA_028408465.1 | idAnaLude1.1 | *Anastrepha ludens* | 820766187 | Chromosome | 2023/2/6 |
| GCA_028414665.1 | idAnaLude1.1 alternate haplotype | *Anastrepha ludens* | 786673534 | Scaffold | 2023/2/2 |
| GCA_029783585.1 | ASM2978358v1 | *Anastrepha ludens* | 988757411 | Scaffold | 2023/4/17 |
| GCF_028408465.1 | idAnaLude1.1 | *Anastrepha ludens* | 820766187 | Chromosome | 2023/2/6 |
| GCA_027943255.1 | idAnaObli1_1.0 | *Anastrepha obliqua* | 836742189 | Chromosome | 2023/1/24 |
| GCA_027943285.1 | idAnaObli1_1.0 | *Anastrepha obliqua* | 843352941 | Contig | 2023/1/23 |
| GCF_027943255.1 | idAnaObli1_1.0 | *Anastrepha obliqua* | 836742189 | Chromosome | 2023/1/24 |
| GCA_027475135.1 | ASM2747513v1 | *Bactrocera correcta* | 702652060 | Chromosome | 2023/1/3 |
| GCA_000789215.2 | ASM78921v2 | *Bactrocera dorsalis* | 414968693 | Scaffold | 2014/12/3 |
| GCA_020283865.1 | ASM2028386v1 | *Bactrocera dorsalis* | 468671466 | Chromosome | 2021/10/6 |
| GCA_023373825.1 | ASM2337382v1 | *Bactrocera dorsalis* | 530310706 | Chromosome | 2022/5/16 |
| GCA_029030885.1 | ASM2903088v1 | *Bactrocera dorsalis* | 524751100 | Contig | 2023/3/8 |
| GCF_000789215.1 | ASM78921v2 | *Bactrocera dorsalis* | 414968693 | Scaffold | 2014/12/3 |
| GCF_023373825.1 | ASM2337382v1 | *Bactrocera dorsalis* | 530310706 | Chromosome | 2022/5/16 |
| GCA_001853355.1 | ASM185335v1 | *Bactrocera latifrons* | 462489382 | Scaffold | 2016/10/25 |
| GCF_001853355.1 | ASM185335v1 | *Bactrocera latifrons* | 462489382 | Scaffold | 2016/10/25 |
| GCA_021035045.1 | ASM2103504v1 | *Bactrocera minax* | 341834660 | Scaffold | 2021/12/3 |
| GCA_021495945.1 | ASM2149594v1 | *Bactrocera minax* | 330472768 | Scaffold | 2022/1/18 |
| GCA_021498325.1 | ASM2149832v1 | *Bactrocera minax* | 323793189 | Contig | 2022/1/18 |
| GCA_029783545.1 | ASM2978354v1 | *Bactrocera minax* | 325327401 | Scaffold | 2023/4/17 |
| GCA_024586455.2 | APGP_CSIRO_Bneo_wtdbg2-racon-allhic-juicebox.fasta_v2 | *Bactrocera neohumeralis* | 577326612 | Chromosome | 2022/8/9 |
| GCF_024586455.1 | APGP_CSIRO_Bneo_wtdbg2-racon-allhic-juicebox.fasta_v2 | *Bactrocera neohumeralis* | 577326612 | Chromosome | 2022/8/9 |
| GCA_001014625.1 | ASM101462v1 | *Bactrocera oleae* | 334371070 | Scaffold | 2015/5/28 |
| GCA_001188975.4 | MU_Boleae_v2 | *Bactrocera oleae* | 484931532 | Scaffold | 2019/4/30 |
| GCF_001188975.3 | MU_Boleae_v2 | *Bactrocera oleae* | 484931532 | Scaffold | 2019/4/30 |
| GCA_000695345.1 | Assembly 2.2 of Bactrocera tryoni genome | *Bactrocera tryoni* | 519005690 | Scaffold | 2014/5/21 |
| GCA_016617805.2 | CSIRO_BtryS06_freeze2 | *Bactrocera tryoni* | 570642875 | Chromosome | 2021/1/19 |
| GCF_016617805.1 | CSIRO_BtryS06_freeze2 | *Bactrocera tryoni* | 570642875 | Chromosome | 2021/1/19 |
| GCA_000347755.4 | Ccap_2.1 | *Ceratitis capitata* | 436474819 | Scaffold | 2017/11/6 |
| GCA_905071925.1 | EGII-3.2.1 | *Ceratitis capitata* | 471266829 | Scaffold | 2020/11/22 |
| GCF_000347755.3 | Ccap_2.1 | *Ceratitis capitata* | 436474819 | Scaffold | 2017/11/6 |
| GCA_024741315.1 | ASM2474131v1 | *Eurosta solidaginis* | 827167314 | Scaffold | 2022/8/24 |
| GCA_029619165.1 | ASM2961916v1 | *Eurosta solidaginis* | 1443056660 | Scaffold | 2023/4/7 |
| GCA_001015115.1 | ASM101511v1 | *Eutreta diana* | 233053157 | Scaffold | 2015/5/29 |
| GCA_029783565.1 | ASM2978356v1 | *Rhagoletis cerasi* | 1153366130 | Scaffold | 2023/4/17 |
| GCA_013731165.1 | Rhpom_1.0 | *Rhagoletis pomonella* | 1223288327 | Scaffold | 2020/6/18 |
| GCF_013731165.1 | Rhpom_1.0 | *Rhagoletis pomonella* | 1223288327 | Scaffold | 2020/6/18 |
| GCA_001687245.2 | Rhagoletis_zephyria_1.1 | *Rhagoletis zephyria* | 1109796908 | Scaffold | 2016/7/19 |
| GCF_001687245.2 | Rhagoletis_zephyria_1.1 | *Rhagoletis zephyria* | 1109796908 | Scaffold | 2016/7/19 |
| GCA_001017515.1 | ASM101751v1 | *Tephritis californica* | 342257700 | Scaffold | 2015/6/1 |
| GCA_001014665.1 | ASM101466v1 | *Trupanea jonesi* | 97281263 | Scaffold | 2015/5/28 |
| GCA_000806345.2 | ASM80634v2 | *Zeugodacus cucurbitae* | 374617745 | Scaffold | 2014/12/23 |
| GCA_028554715.1 | idZeuCucr1.1 alternate haplotype | *Zeugodacus cucurbitae* | 384173189 | Scaffold | 2023/2/8 |
| GCA_028554725.2 | idZeuCucr1.2 | *Zeugodacus cucurbitae* | 439243742 | Chromosome | 2023/2/9 |
| GCF_000806345.2 | ASM80634v2 | *Zeugodacus cucurbitae* | 374617745 | Scaffold | 2014/12/23 |
| GCF_028554725.1 | idZeuCucr1.2 | *Zeugodacus cucurbitae* | 439243742 | Chromosome | 2023/2/9 |

**Table S2** Statistics for sequencing data for *Neoceratitis asiatica* genome assembly

| Method | Insert size (bp) | Data (Gb) | Coverage (X) | Usage |
| --- | --- | --- | --- | --- |
| Illumina NovaSeq | 500 | 31.460 | 55.799 | Survey, correction |
| Nanopore | 20,000 | 74.136 | 131.493 | De novo assembly |
| Hi-C library | 100-500 | 51.876 | 92.011 | Chromosome-level assembly |
| Total | / | 157.472 | 279.303 | / |

**Table S3** Completeness of *Neoceratitis asiatica* genome assembly and annotation evaluated by BUSCO based on insecta_odb10 database (1,367 genes)

| Source | Complete  (C) | Single  copy (S) | Duplicated  (D) | Fragmented  (F) | Missing  (M) |
| --- | --- | --- | --- | --- | --- |
| Contig-level | 99.3% | 98.8% | 0.5% | 0.4% | 0.3% |
| Chromosome-level | 99.1% | 98.7% | 0.4% | 0.3% | 0.6% |
| Annotation | 90.7% | 90.0% | 0.7% | 3.1% | 6.2% |

**Table S4** Summary of Hi-C data for chromosome-level assembly of *Neoceratitis asiatica* genome

| **Parameter** | **Value** |
| --- | --- |
| Sequenced Read Pairs | 171,321,303 |
| Normal Paired | 84,940,663 (49.58%) |
| Chimeric Paired | 67,758,228 (39.55%) |
| Chimeric Ambiguous | 16,843,400 (9.83%) |
| Unmapped | 1,779,012 (1.04%) |
| Ligation Motif Present | 131,406,751 (76.70%) |
| Alignable (Normal+Chimeric Paired) | 152,698,891 (89.13%) |
| Unique Reads | 116,056,271 (67.74%) |
| PCR Duplicates | 35,489,184 (20.71%) |
| Optical Duplicates | 1,153,436 (0.67%) |
| Library Complexity Estimate | 270,784,110 |
| Intra-fragment Reads | 11,292,753 (6.59% / 9.73%) |
| Below MAPQ Threshold | 26,844,604 (15.67% / 23.13%) |
| Hi-C Contacts | 77,918,914 (45.48% / 67.14%) |
| Ligation Motif Present | 65,283,779 (38.11% / 56.25%) |
| 3' Bias (Long Range) | 75% - 25% |
| Pair Type %(L-I-O-R) | 25% - 25% - 25% - 25% |
| Inter-chromosomal | 20,744,185 (12.11% / 17.87%) |
| Intra-chromosomal | 57,174,729 (33.37% / 49.26%) |
| Short Range (<20Kb) | 30,033,242 (17.53% / 25.88%) |
| Long Range (>20Kb) | 27,140,302 (15.84% / 23.39%) |

**Table S5** Statistics for noncoding RNA genes in the genome of *Neoceratitis asiatica*

| **Types** | | **Number** |
| --- | --- | --- |
| **Infernal stats** | Candidate tRNAs read | 3,500 |
|  | Infernal-confirmed tRNAs | 1,368 |
|  | Bases scanned by Infernal | 316,572 |
| **tRNA count** | tRNAs decoding Standard 20 AA | 424 |
|  | Selenocysteine tRNAs (TCA) | 2 |
|  | Possible suppressor tRNAs (CTA,TTA,TCA) | 0 |
|  | tRNAs with undetermined/unknown isotypes | 1 |
|  | Predicted pseudogenes | 941 |
| **Total tRNAs** |  | 1,368 |
| **tRNAs with introns** |  | 29 |
| **rRNA** | 5s ribosomal RNA | 67 |
|  | 5.8s ribosomal RNA | 6 |
|  | Eukaryotic large subunit ribosomal RNA | 10 |
|  | Eukaryotic small subunit ribosomal RNA | 3 |
| **miRNA** |  | 69 |
| **lncRNA** |  | 2 |
| **sRNA** |  | 3 |
| **snRNA** |  | 74 |

**Table S6** Statistics for repeat elements in the genome of *Neoceratitis asiatica*

| **Types** | **Number** | **Length (bp)** | **Percentage (%)** |
| --- | --- | --- | --- |
| Retroelements | 371986 | 127426370 | 22.7 |
| SINEs | 28659 | 5316445 | 0.95 |
| Penelope | 2169 | 264828 | 0.05 |
| LINEs | 252481 | 75730209 | 13.49 |
| CRE/SLACS | 0 | 0 | 0 |
| L2/CR1/Rex | 22481 | 8689177 | 1.55 |
| R1/LOA/Jockey | 22591 | 6430095 | 1.15 |
| R2/R4/NeSL | 3419 | 1385393 | 0.25 |
| RTE/Bov-B | 111884 | 32852010 | 5.85 |
| L1/CIN4 | 439 | 22254 | 0 |
| LTR elements | 90846 | 46379716 | 8.26 |
| BEL/Pao | 27544 | 11665448 | 2.08 |
| Ty1/Copia | 1455 | 1273550 | 0.23 |
| Gypsy/DIRS1 | 44211 | 28378637 | 5.06 |
| Retroviral | 1001 | 422711 | 0.08 |
| DNA transposons | 264341 | 94832601 | 16.9 |
| hobo-Activator | 6997 | 1434799 | 0.26 |
| Tc1-IS630-Pogo | 191559 | 76929310 | 13.71 |
| En-Spm | 0 | 0 | 0 |
| MuDR-IS905 | 0 | 0 | 0 |
| PiggyBac | 2008 | 1010576 | 0.18 |
| Tourist/Harbinger | 2979 | 702669 | 0.13 |
| Other (Mirage, P-element, Transib) | 2256 | 803623 | 0.14 |
| Rolling-circles | 44899 | 8932318 | 1.59 |
| Unclassified | 230656 | 37225574 | 6.63 |
| Total interspersed repeats |  | 259484545 | 46.23 |
| Small RNA | 0 | 0 | 0 |
| Satellites | 64 | 47039 | 0.01 |
| Simple repeats | 283449 | 20246392 | 3.61 |
| Low complexity | 51371 | 6811353 | 1.21 |

**Table S7** Statistics of comparative genomics

| **Types** | **Number** |
| --- | --- |
| Number of species | 10 |
| Number of genes | 257702 |
| Number of genes in orthogroups | 246374 |
| Number of unassigned genes | 11328 |
| Percentage of genes in orthogroups | 95.6 |
| Percentage of unassigned genes | 4.4 |
| Number of orthogroups | 17735 |
| Number of species-specific orthogroups | 3136 |
| Number of genes in species-specific orthogroups | 11958 |
| Percentage of genes in species-specific orthogroups | 4.6 |
| Mean orthogroup size | 13.9 |
| Median orthogroup size | 11 |
| G50 (assigned genes) | 19 |
| G50 (all genes) | 18 |
| O50 (assigned genes) | 3474 |
| O50 (all genes) | 3777 |
| Number of orthogroups with all species present | 7695 |
| Number of single-copy orthogroups | 613 |

**Table S8** Gene orthology comparison involving 10 insect species

| **Common name** | **Species code** | **1:1:1** | **N:N:N** | **Mono** | **Poly** | **SD** | **ND** | **UG** |
| --- | --- | --- | --- | --- | --- | --- | --- | --- |
| Monophagous flies | Nasi | 613 | 16537 | 2543 | 0 | 926 | 10399 | 1237 |
|  | Bole | 613 | 32719 | 200 | 0 | 5433 | 11598 | 394 |
|  | Rzep | 613 | 28658 | 2376 | 0 | 6499 | 13765 | 5316 |
| Polyphagous flies | Ccap | 613 | 22093 | 0 | 206 | 4345 | 11499 | 243 |
|  | Bdor | 613 | 14249 | 0 | 446 | 1429 | 11978 | 913 |
|  | Blat | 613 | 21881 | 0 | 74 | 4349 | 11644 | 144 |
|  | Btry | 613 | 23190 | 0 | 179 | 4398 | 11882 | 147 |
|  | Zcuc | 613 | 23395 | 0 | 394 | 4414 | 11689 | 207 |
|  | Rpom | 613 | 29296 | 0 | 1422 | 6738 | 13062 | 849 |
|  | Dmel | 613 | 28226 | 0 | 4118 | 7256 | 11273 | 1472 |

Note: "1:1:1” indicates single-copy genes in all species; “N:N:N” indicates multi-copy genes in all species; “Mono” indicates monophagous fly-specific genes; “Poly” indicates polyphagous fly-specific genes; “SD” indicates species-specific duplicated genes; “ND” indicates species-specific genes; “UG” indicates unassigned genes.

**Table S9** Statistics of gene expansion and contraction

| **Species** | **Expanded fams** | **Genes gained** | **genes/expansion** | **Contracted fams** | **Genes lost** | **genes/contraction** | **No change** | **Avg. Expansion** |
| --- | --- | --- | --- | --- | --- | --- | --- | --- |
| Bole | 3824 (2618) | 12772 | 3.34 | 1960 (163) | 2242 | 1.14 | 11951 | 0.593741 |
| Btry | 2052 (1857) | 4241 | 2.07 | 1706 (1079) | 1833 | 1.07 | 13977 | 0.135777 |
| Rzep | 3439 (2956) | 6646 | 1.93 | 1971 (1728) | 2595 | 1.32 | 12325 | 0.228418 |
| Bdor | 715 (614) | 1378 | 1.93 | 4252 (2283) | 7911 | 1.86 | 12768 | -0.36837 |
| Ccap | 2375 (1417) | 4751 | 2 | 826 (38) | 910 | 1.1 | 14534 | 0.216577 |
| Nasi | 439 (375) | 5955 | 13.56 | 4527 (1495) | 7670 | 1.69 | 12769 | -0.0967 |
| Rpom | 3806 (3136) | 7738 | 2.03 | 2517 (2245) | 3049 | 1.21 | 11412 | 0.264392 |
| Blat | 2033 (1797) | 3525 | 1.73 | 2059 (1390) | 2426 | 1.18 | 13643 | 0.061968 |
| Zcuc | 2040 (1098) | 4023 | 1.97 | 2476 (176) | 2750 | 1.11 | 13219 | 0.071779 |
| Dmel | 3845 (242) | 6260 | 1.63 | 7439 (324) | 8087 | 1.09 | 6451 | -0.10302 |


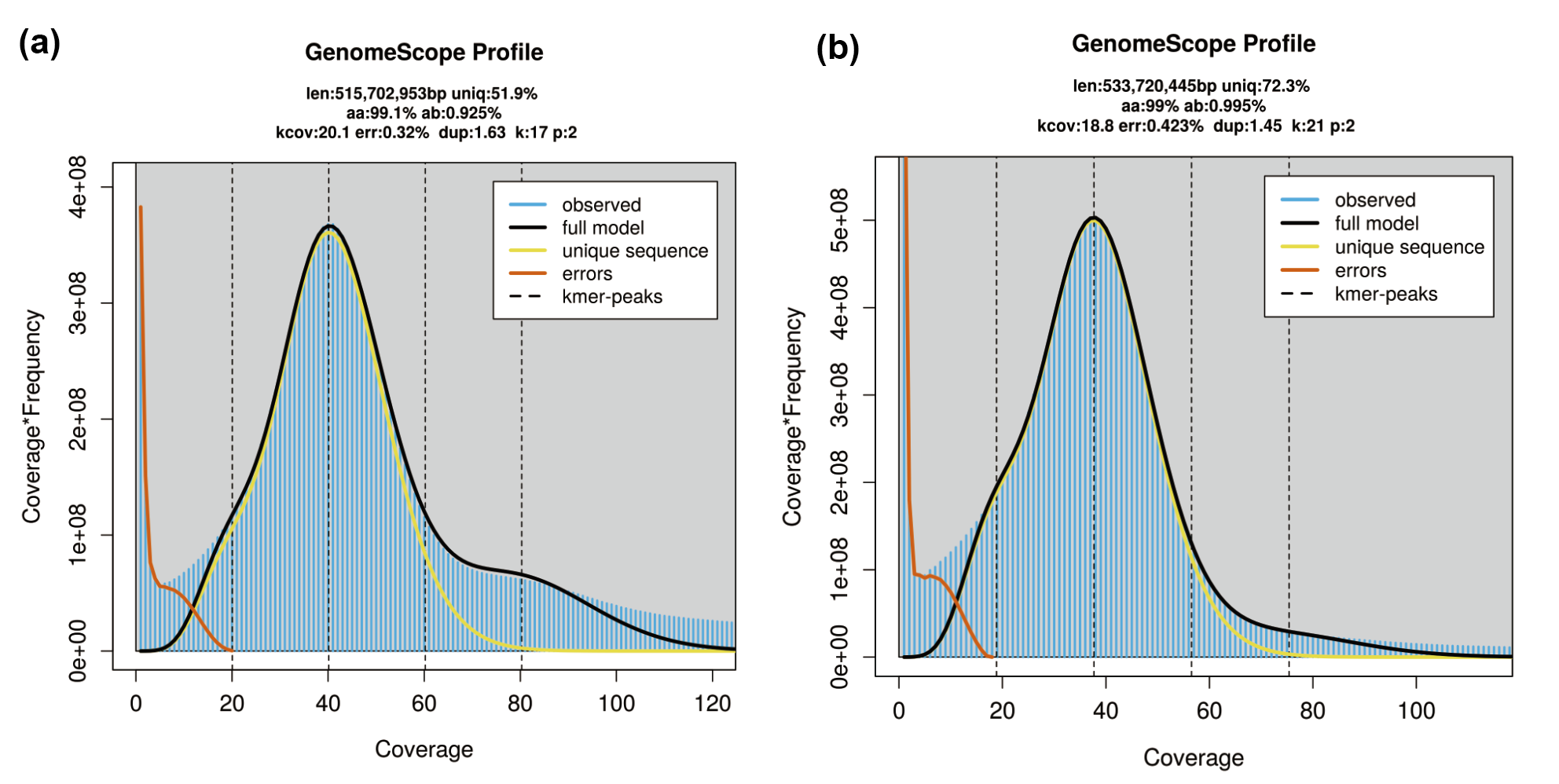


**Fig. S1** Assembly features of *Neoceratitis asiatica* genome. Genome size, heterozygosity, and duplication rate of the genome were estimated by the K-mer method with 17-mer (a) and 21-mer (b).


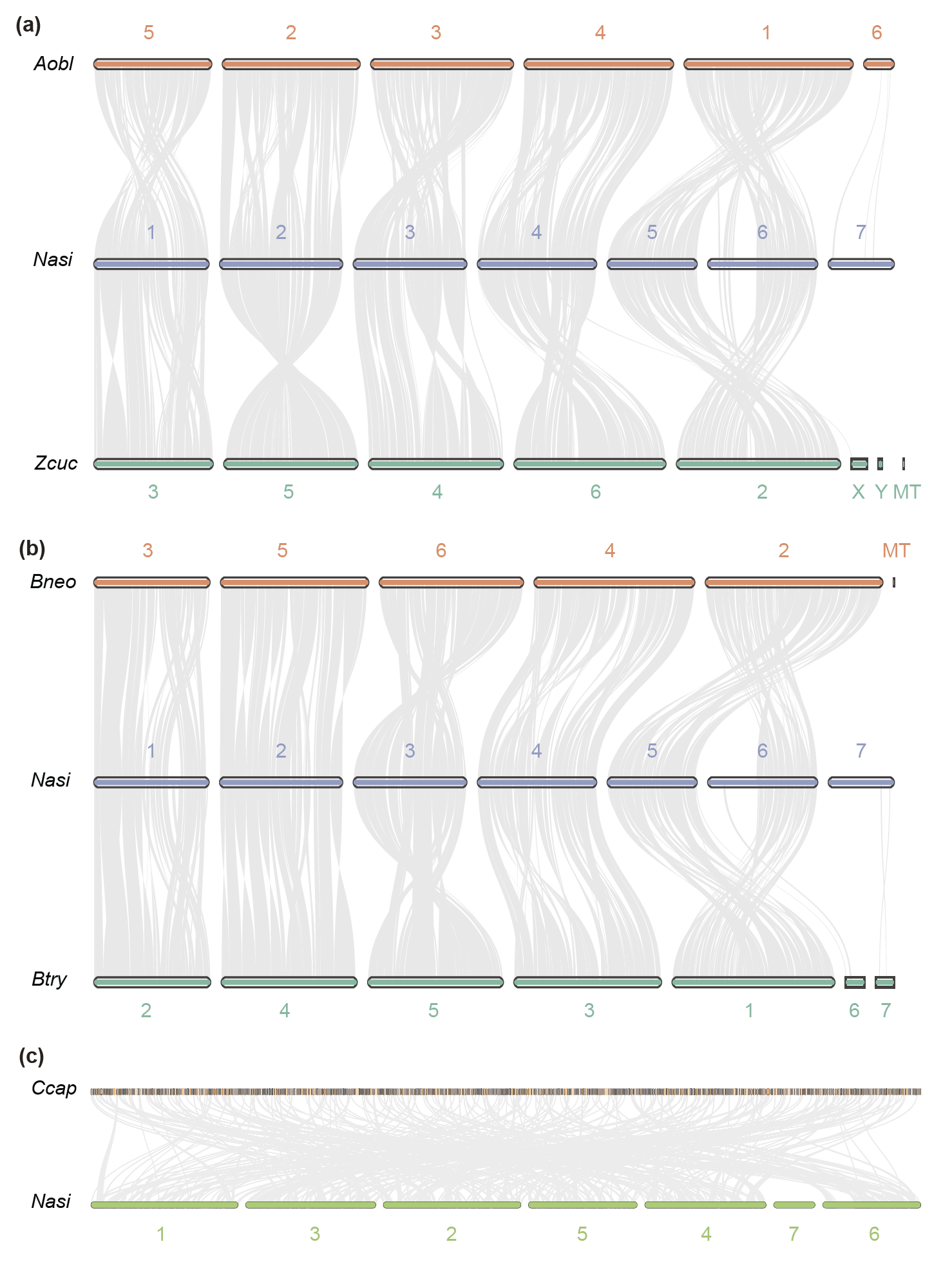


**Fig. S2** Synteny blocks among genomes of different flies. The extensive synteny among *Neoceratitis asiatica* (*Nasi*), *Anastrepha obliqua* (*Aobl*) and *Zeugodacus cucurbitae* (*Zcuc*) (a); among *Neoceratitis asiatica* (*Nasi*), *Bactrocera neohumeralis* (*Bneo*) and *Bactrocera tryoni* (*Btry*) (b); between *Neoceratitis asiatica* (*Nasi*) and *Ceratitis capitata* (*Ccap*).


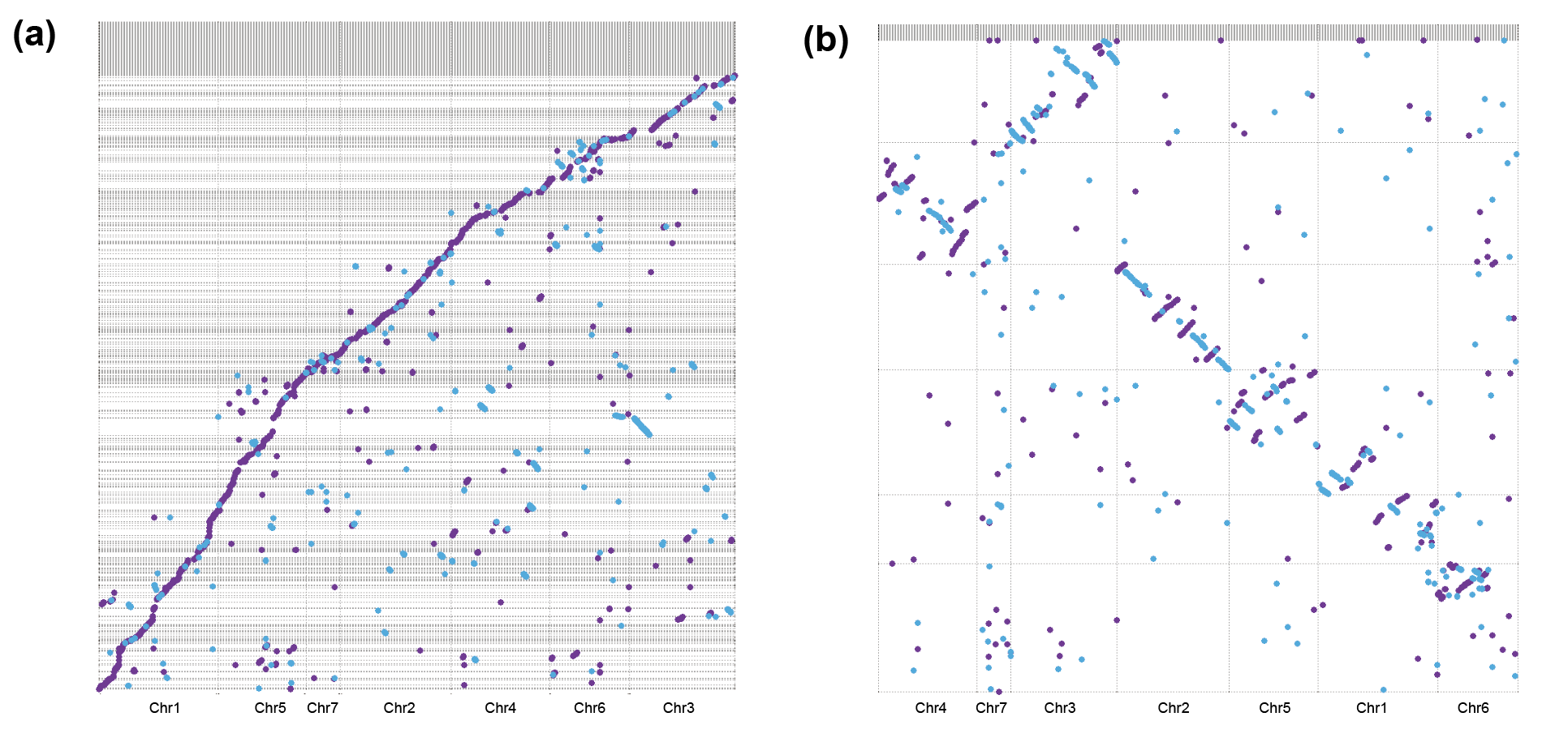
**Fig. S3** Alignment of large fragment sequences between Tephritidae flies. There is higher similarity in the chromosome structure between the *Neoceratitis asiatica* and *Ceratitis capitata* (a), while significant differences between *Neoceratitis asiatica* and *Bactrocera dorsalis* (b).


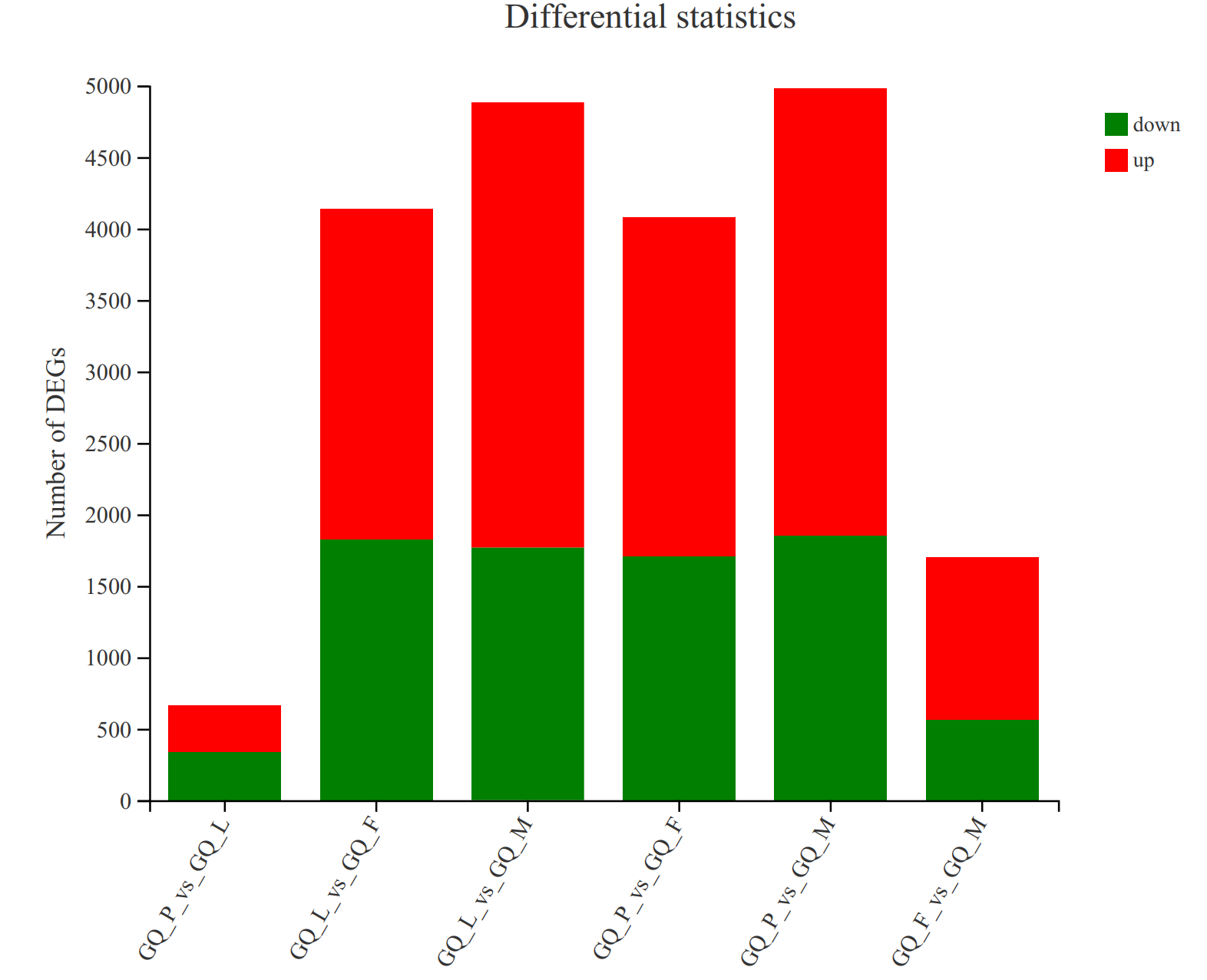


**Fig. S4** The number of differentially expressed genes (DEGs) among different developmental stages in *Neoceratitis asiatica.*
